# Ethical and scientific considerations on the establishment of a controlled human infection model for schistosomiasis in Uganda: report of a stakeholders’ meeting held in Entebbe, Uganda.

**DOI:** 10.12688/aasopenres.12841.2

**Published:** 2018-08-06

**Authors:** Alison M. Elliott, Meta Roestenberg, Anne Wajja, Christopher Opio, Francis Angumya, Moses Adriko, Moses Egesa, Serah Gitome, Joseph Mfutso-Bengo, Philip Bejon, Melissa Kapulu, Zoe Seager, Tom Lutalo, Winfred Badanga Nazziwa, Asuman Muwumuza, Maria Yazdanbakhsh, Pontiano Kaleebu, Narcis Kabatereine, Edridah Tukahebwa

**Affiliations:** 1 Medical Research Council/Uganda Virus Research Institute and London School of Hygiene & Tropical Medicine (MRC/UVRI and LSHTM) Uganda Research Unit, Entebbe, P.O. Box 49, Uganda; 2 Department of Parasitology, Leiden University Medical Center, Leiden, The Netherlands; 3 Department of Medicine, College of Health Sciences, Makerere University, Kampala, P.O. Box 7072, Uganda; 4 Vector Control Division, Ministry of Health of Uganda, Kampala, Uganda; 5 Department of Medical Microbiology, School of Biomedical Sciences, College of Health Sciences, Makerere University, Kampala, Uganda; 6 Kenya Medical Research Institute (KEMRI), Nairobi, Kenya; 7 Centre for Bioethics for Eastern and Southern Africa, School of Public Health and Family Medicine, College of Medicine, University of Malawi, Blantyre, Malawi; 8 KEMRI-Wellcome Trust Research Programme, Kilifi, Kenya; 9 Wellcome Trust, London, UK; 10 Uganda Virus Research Institute, Entebbe, Uganda; 11 Uganda National Council for Science and Technology, Kampala, Uganda; 12 Mukono District Local Government, Mukono, Uganda; 13 Schistosomiasis Control Initiative, Faculty of Medicine, School of Public Health, Imperial College London, London, UK

**Keywords:** Controlled human infection model; Schistosoma mansoni; Uganda; The Netherlands

## Abstract

Controlled human infection (CHI) models are gaining recognition as an approach to accelerating vaccine development, for use in both non-endemic and endemic populations: they can facilitate identification of the most promising candidate vaccines for further trials and advance understanding of protective immunity. Helminths present a continuing health burden in sub-Saharan Africa. Vaccine development for these complex organisms is particularly challenging, partly because protective responses are akin to mechanisms of allergy. A CHI model for *Schistosoma mansoni *(CHI-S) has been developed at Leiden University Medical Centre, the Netherlands. However, responses to schistosome infections, and candidate vaccines, are likely to be different among people from endemic settings compared to schistosome-naïve Dutch volunteers. Furthermore, among volunteers from endemic regions who have acquired immune responses through prior exposure, schistosome challenge can be used to define responses associated with clinical protection, and thus to guide vaccine development.  To explore the possibility of establishing the CHI-S in Uganda, a Stakeholders’ Meeting was held in Entebbe in 2017. Regulators, community members, researchers and policy-makers discussed implementation challenges and recommended preparatory steps: risk assessment; development of infrastructure and technical capacity to produce the infectious challenge material in Uganda; community engagement from Parliamentary to grass-roots level; pilot studies to establish approaches to assuring fully informed consent and true voluntariness, and strategies for selection of volunteers who can avoid natural infection during the 12-week CHI-S; the building of regulatory capacity; and the development of study protocols and a product dossier in close consultation with ethical and regulatory partners. It was recommended that, on completion, the protocol and product dossier be reviewed for approval in a joint meeting combining ethical, regulatory and environment management authorities. Most importantly, representatives of schistosomiasis-affected communities emphasised the urgent need for an effective vaccine and urged the research community not to delay in the development process.

## Introduction

Effective vaccines have proven extremely useful in the prevention of infectious diseases, but are still lacking for major poverty-related and neglected infections, including helminth infections. The conventional approach to vaccine development, testing efficacy in human subjects in large Phase III trials after safety and immunogenicity are confirmed through smaller Phase I and II trials, is lengthy and extremely costly. An alternative approach, to identify the most promising candidate vaccines through controlled human infection (CHI) models (typically referred to as Phase IIa), is gaining acceptance and application for infections including malaria, typhoid and others: to date about 22,000 volunteers have been infected, safely, with 23 different pathogens ^1– 4^.

Schistosomiasis is a major parasitic infectious disease, considered second only to malaria as a parasitic cause of morbidity and mortality ^5^. The current approach to control schistosomiasis is through mass drug administration (MDA) with praziquantel, but this is limited by high rates of re-infection and there are concerns about the possible emergence of drug resistance ^6, 7^. An effective vaccine would be an extremely valuable control tool but vaccine development for this complex organism is challenging. In a bid to accelerate this, Meta Roestenberg and colleagues at the Leiden University Medical Centre have developed a controlled human infection model for *Schistosoma mansoni* (CHI-S) and tested it among Dutch volunteers. However, the response to *Schistosoma* infection, and to candidate vaccines, is likely to differ markedly among people from endemic African populations (where vaccines are most needed and where people are exposed to an abundance of potentially immunomodulating infections) compared to European volunteers. Furthermore, individuals from endemic populations may display some resistance to CHI-S due to prior schistosome exposure. Vaccine development against several pathogens has been informed by studies in which naturally acquired immune responses are correlated with clinical protection, in order to inform vaccine developers on ideal antigens, epitopes and protective thresholds. Thus challenge studies among volunteers from endemic settings, who have naturally acquired immunity, have the potential also to accelerate the development of the next generation of vaccines by allowing desirable immune responses to be identified and prioritised. Implementation of the CHI-S model in an endemic setting would therefore provide critical additional information on markers of protective immunity and on immunogenicity, safety and efficacy of candidate vaccines. 

As a first step towards establishing the CHI-S in an endemic setting, we held a stakeholders meeting in Entebbe, Uganda, in November 2017, to identify key challenges and to develop strategies to address them. Meeting participants included representatives of Uganda’s Ministry of Health (Vector Control Division), National Council for Science and Technology, National Drug Authority and National Environment Management Authority; researchers and clinicians who manage schistosomiasis and its complications; chairpersons, committee members and community representatives from various Ugandan ethics fora across the country (the Uganda Virus Research Institute, Makerere University and Mbarara University); representatives of potential volunteer communities (Makerere University students and community representatives from Koome Islands in Lake Victoria); colleagues with experience of implementing controlled human malaria infections (CHMI) from Kenya and with ethics expertise from Kenya and Malawi; and the team who developed the CHI-S from Leiden. Deliberations were informed by the earlier work on CHMI in Kenya, and by the proceedings of the meeting on CHI models held in Malawi in June 2017 ^8^. We here report proceedings of the Uganda meeting.

## Schistosomiasis

Schistosomiasis is estimated to affect 230 million people worldwide, the majority of them in sub-Saharan Africa ^9^. In Uganda, schistosomiasis was first described in the 1900s and was recognised as a serious public health problem in the 1950s ^10^. Mapping showed the distribution of infection around the major lakes and rivers, and peak intensity among children and adolescents in the five to 20 year old age range ^11^. The development of a control plan in the 1990s, provided a strong basis for the work of the Schistosomiasis Control Initiative, which launched its programme of control by Mass Drug Administration using praziquantel in Uganda in 2003. Initial results from MDA were promising ^12^ but recent data show that, despite enhanced coverage, both prevalence and intensity of infection remain high, especially among school-age children, in “hot spot” lakeshore, hard-to reach (such as island) communities. It is increasingly evident that MDA alone will not be adequate to achieve WHO’s target of elimination of schistosomiasis as a public health problem by 2030. Of Uganda’s population of 36 million, more than 4 million are estimated to be infected with schistosomiasis, and 55% of the present population is estimated to be at risk ^13^. 

**Figure 1.**
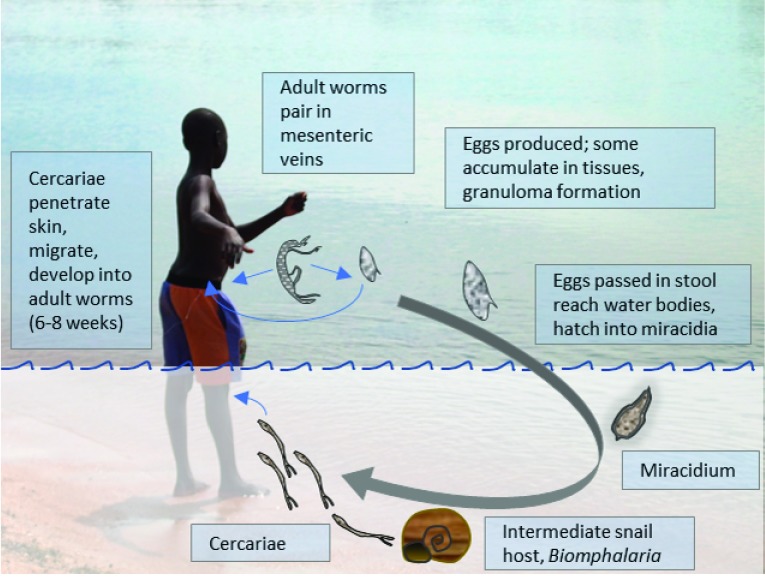
The life cycle of *Schistosoma mansoni*. During natural infection, individuals are usually infected with multiple cercariae, both male and female, which mature into adults, pair in the mesenteric blood vessels, and produce eggs, the main cause of pathology. In the controlled human infection model, single sex (male) cercariae are used to avoid the development of eggs and consequent pathology.

Adult *Schistosoma* worms reside in blood vessels around the gut ( *S. mansoni*, *S. intercalatum* and *S. japonicum*) or urinary bladder ( *S. haematobium*), where the female lays eggs which are excreted through the intestinal or bladder wall and voided in stool or urine. In water, each egg hatches producing a single miracidium. This enters the intermediate snail host where it multiplies asexually, producing identical cercariae. Cercariae are shed into the water where they again infect the human host by penetrating through the skin ( Figure 1) ^9^. 

Humans sometimes experience cercarial dermatitis in response to the penetrating parasites and a minority develop acute schistosomiasis syndrome (“Katayama Fever”) in reaction to an initial infection. However, most serious disease caused by schistosome infection is due to the eggs. Besides being excreted in stool or urine, many eggs also find their way into other tissues, notably the liver: progressive liver fibrosis results in portal hypertension, splenomegaly, and ascites; oesophageal varices develop which can lead to death through uncontrolled haemorrhage ^9^. Effective management, for example by repeated, endoscopic band ligation of the varices ^14^, is seldom available in the resource-limited settings where schistosomiasis is common: upper gastrointestinal bleeding, resulting from *S. mansoni*-induced periportal fibrosis is a common complaint in primary health care in Northern Uganda, along the course of the Nile ^15, 16^. Hepatosplenic schistosomiasis is also associated with stunted growth and anaemia, leucopenia and thrombocytopenia. Occasionally eggs can be found in the spinal cord or brain, causing neuropathology ^9^. 

## Vaccines for schistosomiasis

A vaccine for schistosomiasis has been ranked among the top 10 vaccines that need to be developed urgently ^17^. Schistosomes are large, multicellular animals that have evolved to co-exist with their human host. The immunoregulatory properties of schistosomes, which enable them to live in the portal vasculature without immune clearance, are likely to impede vaccine development ^18^. The complex interplay between T-helper (Th)1, Th2 and regulatory responses is still incompletely understood. Schistosome killing is mediated by antibody responses, and particularly by Immunoglobulin (Ig)E ^19^, presenting the risk that an effective IgE-inducing vaccine might induce allergic reactions, especially among previously-exposed individuals from endemic populations (as in the case of a candidate hookworm vaccine ^20^). T-helper (Th)2 response profiles are therefore undesirable. Th1 responses must be targeted. In animal models a Th1 response has been shown to be able to participate in immunity against schistosomes ^21^, but it is not yet certain which Th1 responses can induce protective immunity and correlates of protection have not been identified. An ideal anti-schistosome vaccine would be suitable for use among young children in endemic settings, given their high burden of infection, as well as adults in high-risk occupations; it would achieve 75% reduction in infection intensity (assessed by circulating antigen or egg production); it would require administration of, at most, two doses; it would induce protection lasting at least five to 10 years ^22^; it would not induce IgE; and it would be suitable to co-administer with MDA.

Attenuated whole organisms from some helminth species, including schistosome cercariae, have been shown to induce protective immunity in animals ^23, 24^, but production of attenuated cercariae for large-scale administration is not feasible, thus the current goal is to identify helminth antigens that induce protective responses, but not IgE. Approaches to this include the use of sera generated in animal studies using attenuated larvae, or from human population studies that determine resistance to re-infection after MDA, together with recombinant antigens developed from the investigation of the parasite transcriptome and proteome, to identify antigen- and stage-specific antibodies associated with protection ^25, 26^. To date, four antigens (SmTSP-2, Sm14, Smp80 and Sh28GST) have been identified and tested, and show promise of efficacy in animal models; three (SmTSP-2, Sm14, Smp80) are ready to enter Phase I trials and one (Sh28GST, Bilhvax) has undergone a Phase III trial ( NCT00870649: this has been completed but no data have yet been released on the outcome of the trial). However, transcriptomic-proteomic approaches suggest many more candidates that could be evaluated as vaccine antigens, either singly or in combinations ^27– 29^. Unfortunately, the limited resources available for schistosome vaccine development restrict the number of candidates that can be taken forward. As yet, the value of animal models for predicting efficacy of, and responses to, schistosome vaccine candidates in humans is unknown. Murine models may not be the optimal platform; baboons are considered the most suitable, and have been used to further the SmTSP-2 vaccine candidate to phase I testing in humans, but they are expensive and reagents for immunological studies are limited. In general, animal models have been of great utility in asking fundamental questions regarding immunology and demonstrating proof-of-principle of particular vaccination strategies, but do not recapitulate precisely the physiology of human infections, and therefore cannot be considered a substitute for human studies. Clinical testing of novel candidates in humans is needed to obtain true efficacy data.

## The controlled human schistosome infection model

The CHI-S model addresses many of the roadblocks to development of an effective vaccine for schistosomiasis ( Table 1). The model alone will provide novel information on the evolution of immune responses following infection. Combined with a model “vaccine”, such as irradiated cercariae, it has the potential to identify correlates of protection, particularly protective Th1 responses that could be harnessed for vaccine development. Then, used as a challenge in phase I vaccine trials, CHI-S will allow efficient and timely selection of potential vaccine candidates, and hence could improve and accelerate the vaccine development pipeline. Implemented in the endemic setting, CHI-S will take into account the impact of prior and current exposure to schistosome infection (including pre-natal exposure) ^30^ and allow modelling of efficacy in target populations, and in association with praziquantel MDA. In addition, CHI-S offers potential for testing the efficacy of new drugs for treatment of schistosomiasis. 

**Table 1.** Road blocks to schistosome vaccine development and how controlled human infection models for schistosomiasis can help. (CHI-S) - Controlled human infection model for *Schistosoma mansoni*.

Road blocks		How CHI-S can help
**Vaccine candidates:** Several vaccine candidates are available; but • there are limited resources to take candidates forward • the focal and dynamic epidemiology of schistosomiasis may make efficacy studies difficult • the requirement for pretreatment of subjects before vaccine testing can influence epidemiology and complicate the efficacy evaluation	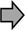	✓ CHI-S quickly identifies candidates most likely to induce protection ✓ CHI-S can be performed in populations with defined pre-exposure
**Animal models:** Suitability of various animals for predicting responses to, and efficacy of, vaccine candidates in humans not known	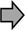	✓ CHI-S provides direct evidence of responses in humans
**Immunological road-blocks:** • Schistosomes induce regulatory responses which could impair vaccine immunogenicity • Schistosomes induce Th2 responses and IgE with accompanying risk of allergic phenomena • Th1 responses involved in protection not known in humans • Correlates of protection not known	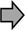	✓ CHI-S describes evolution of immune responses following infection ✓ Combined with a model “vaccine” (such as irradiated cercariae, predicted to be effective) CHI-S identifies protective Th1 responses and correlates of protection

The Leiden CHI-S has been developed with detailed attention to safety in both production and administration of the infectious challenge product. Because eggs are the main source of morbidity and pathology, the CHI-S avoids permanent pathology by making use of single-sex infections. Using the laboratory lifecycle, individual snails are isolated and each snail is carefully infected with a single miracidium of *Schistosoma mansoni*. This undergoes asexual reproduction in the snail and after five weeks produces thousands of cercariae of a single clone, and hence single sex. Following several quality control steps and determination of male or female sex by PCR, male cercariae are used for the controlled infection of volunteers. This is done by taping a chamber of water containing a predetermined number of male cercariae onto the volunteer’s forearm for a 30-minute interval. Work towards an infection model using female cercariae is in progress, but male worms do better than females when not in pairs and the possibility of production of sterile eggs from females needs to be excluded. Successful infections can be detected (usually after six to 12 weeks) and quantified by measuring circulating anodic antigen (CAA) levels in the blood: this is a protein which is secreted into the blood in large quantities by adult worms ^31^. Volunteers are followed up for 12 weeks and then treated with praziquantel. The infected snails and preparation of cercariae are managed in customised, dedicated facilities following Good Manufacturing Practice guidelines. The volunteers are intentionally infected with the male cercariae, and followed up until after they are cured, under conditions analogous to a Phase I Clinical Trial.

The current CHI-S model from Leiden provides a blueprint for developing a model for *S. haematobium (Sh)* also. The need for a *S. haematobium* model, in addition to the existing *S. mansoni* model, will need to be evaluated from a vaccine pipeline perspective.

## Considerations on implementation of the novel CHI-S model in the endemic setting in Uganda

Uganda is well-placed to host the first CHI-S in an endemic setting. Endemicity of *S. mansoni* is high. The schistosomiasis control programme, under the Vector Control Division of the Ministry of Health, has long been a key collaborator in world-leading schistosomiasis research and supports the CHI-S concept. There are good laboratory facilities and there is expertise in maintaining the *S. mansoni* laboratory life-cycle, as well as in molecular and immunological work. There is strong experience of community engagement, and expertise in clinical trials, complemented by an open and engaged ethical and regulatory environment.

At the Uganda Stakeholders’ Meeting reported here, key challenges were identified with regards to the technical procedures (around importation and, or, local production of cercariae for challenge and related risk assessment); community engagement and participant recruitment (around ensuring awareness and full understanding of study procedures and management of the potential for natural exposure during challenge experiments); and ethical as well as regulatory processes (around development of regulatory capacity, documentation and risk assessments). Details of discussions follow, and are summarised in Table 3.

## Technical considerations for implementation of the CHI-S in Uganda

Technical considerations for implementation of the CHI-S in Uganda principally comprise the preparation of cercariae for the inoculum. Because the shelf-life of cercariae is just two hours, cercarial production for human challenge must be done locally. In Leiden, the *S. mansoni* life cycle is maintained in hamsters using a laboratory strain of *S. mansoni* which originated in Puerto Rico and *Biomphalaria glabrata* snails; this snail species is not endemic in Uganda. In Uganda, the laboratory life cycle has previously been maintained by the Vector Control Division of the Ministry of Health for another project, using mice and a range of endemic *Biomphalaria* species (including *B. choanomphala*, *B. stanleyi*, and *B. sudanica*) ^32^, but it is not actively maintained at present. 

Options for preparing the inoculum in Uganda include (1) re-establishing the full *S. mansoni* laboratory life-cycle; (2) shipping cryopreserved miracidia or eggs from Leiden for snail infection and cercarial shedding in Entebbe (technologies for cryopreservation of miracidia or eggs still need to be developed); (3) shipping infected snails from Leiden for shedding in Entebbe.

The third option, of shipping infected snails, is currently the most feasible. Guidelines for shipping live snails (including infected snails), developed by the Danish Bilharzia Laboratory, are available. These will need to be combined with International Air Transport Association (IATA) requirements for shipping of infectious material. It will be important to work with customs officials and handling agents to ensure efficient release on arrival in Uganda. This process will need to be piloted. A risk assessment will need to be undertaken, in collaboration with the Uganda National Environment Management Authority (NEMA) regarding potential introduction of a new snail species and *S. mansoni* strain into Ugandan water bodies and risk management protocols will need to be implemented to ensure that this does not occur. Facilities for housing and shedding the snails, and preparing the inoculum in accordance with GMP guidelines, will need to be established. 

Post-meeting, a fourth option for a truly-local Ugandan CHI-S was proposed. This would involve generating the inoculum by obtaining miracidia from stool samples of infected people in Uganda, and using these to infect snails of a local species in the laboratory. This would have advantages. The use of a non-endemic *Schistosoma* strain and of non-endemic snail species would be avoided, which would reduce the environmental risk involved with quarantined non-endemic snail and schistosome species. In addition, the model would be closer to field infections and thus might be considered more representative of endemic infections in Uganda. Additional capacity would be built in-country. However, this approach would also bring additional challenges. The full life-cycle (option (1) above) would need to be re-established in order to test the Ugandan schistosome strain obtained for praziquantel susceptibility and bioburden before use in controlled human infections. Also, the CHI-S model would need to be validated again, and a dose-finding study to identify the optimal balance between tolerability and attack rate would need to be performed. With this truly-local, Ugandan model, it would be more difficult to interpret any differences in responses to vaccines or to infection between studies in Uganda and studies in Leiden (or elsewhere). Work in CHI models of other pathogens has indicated substantial advantages to standardization of the CHI models across sites to ensure comparability of results. Nevertheless, this remains an important option for further discussion.

Good clinical laboratory practice (GCLP) accredited facilities and expertise for PCR (to confirm male sex of cercariae) are already available in Uganda, and plans are in place to provide equipment for high-sensitivity detection of infection by measurement of serum CAA in 2018. Because these assays represent a critical part of the quality control of the product and the primary endpoint of the trial, they will need to be validated extensively before the trial can start. Immunological expertise for the conduct of antibody ELISAs and cellular immune response assays is also available. However, training of the Ugandan team to undertake specific procedures, and to replicate quality control procedures that have been developed in Leiden, will be key.

## Protocol development and participant recruitment considerations for CHI-S in Uganda

Ugandan researchers have substantial experience of community engagement and of conducting Phase I trials under Good Clinical Practice (GCP) conditions. However, the stakeholders’ meeting recognised that enhanced attention to aspects of these activities would be required for the CHI-S. 

Full details of community engagement plans will be needed as part of the CHI-S protocol. There is need to involve opinion leaders, including members of Parliament such as the Parliamentary Committee on Health, and Resident District Commissioners and District Health Officers of the participating districts, as well as local council leaders, in order to prevent circulation of misinformation about the work. Appropriate information strategies as well as mitigation plans will be put in place to identify any miscommunication on platforms such as social media. Populations of interest for CHI-S will include Ugandans not previously exposed to schistosomiasis (perhaps from an urban setting) as well as those from schistosomiasis-endemic communities (prior exposure for inclusion or exclusion can be determined by measuring IgG antibody to schistosome egg antigen). Experience in Kenya with the controlled human malaria infection (CHMI) model showed that participants with different exposure profiles responded differently to the CHI: participants coming from areas with no active transmission (Nairobi residents) had low baseline responses to malaria, and a challenge response similar to Europeans ^3^, whereas those resident where active malaria transmission occurs had higher baseline responses and a distinct profile of response to challenge (Kapulu, Bejon personal communication).

Kenyan researchers involved university communities for their first CHMI studies ^33^, but a few attendees of the Uganda meeting expressed concern about specifically targeting students. Although adults, university students are often still dependents and parents might have objections. It was agreed that volunteers would be expected to inform their next of kin about their participation and that contact details for the next of kin must be provided in case of emergency. Critical to recruitment, and to obtaining informed consent, would be the inclusion of a test that clearly demonstrates a full understanding of the CHI-S model, and reassurance that participation is truly voluntary. Based on Leiden experience, the time taken by the team to know the potential volunteers, during the initial screening and recruitment procedures, is expected to be valuable in selecting those that will understand, and reliably comply with, the procedures.

Volunteers from endemic communities are likely to be actively infected with *S. mansoni* at the time of recruitment. Such infections will need to be treated with praziquantel before enrolment in the CHI-S. This may require more than one dose of treatment; cure can be determined using the highly-sensitive CAA assay. For individuals from endemic communities, there is also a substantial risk of re-exposure during the 12-week follow-up period between the CHI-S infection and cure; natural infection may be added to the CHI-S infection. While the resulting risk to the volunteer would be comparable to their usual lifestyle, this would invalidate the results of the study. Therefore, volunteers will need to be carefully selected to ensure that they are able and willing to avoid re-exposure. The 12-week duration of the CHI-S follow up means that admission to a facility (as practiced in some CHMI studies) would not be feasible. Follow up of a randomised, placebo group could be considered in order to assess whether there are substantial re-infection rates in a study group. 

The Uganda CHI-S protocol will be expected to meet all the requirements of a phase I trial. A data and safety monitoring board (DSMB) will be needed, as well as internal and external monitoring. A realistic evaluation of risks to the volunteers must be included: the intensity of the risk is expected to be lower than for malaria (for example), such that hospital admission will not be necessary, but a 24-hour helpline will be needed. Treatability and methods of treatment of likely side effects and safety evaluations to be conducted must be mentioned. Insurance provision will be necessary: post-meeting information indicated that this should be provided by a local company or agent, but it was recognised that local insurance companies in Uganda are unfamiliar with clinical trials and education of these bodies is needed. Material transfer agreements will be required for protocols involving import of snails or miracidia, and for export of samples if assays are to be done outside Uganda; data sharing agreements, where necessary, would need to be developed and implemented.

Rates for compensation of lost time or income, and transport costs, to volunteers will need to be specified and it will be challenging to set amounts that recognise demands upon the volunteers but do not constitute an undue inducement, since almost any payment may be an inducement in Ugandan settings. Principles for setting the payments will include estimates of time and income loss from visits and reimbursement for transport costs and other inconveniences. Time compensation should be adjusted to the average income or wages in a particular community (for example the Kenyan CHMI studies offered higher rates of compensation in Nairobi than in Kilifi, based on the premise that Nairobi was an urban setting with higher income than in the coastal town of Kilifi which is in a rural setting). It is generally considered that participants should not be compensated for risk, since this could be interpreted as an undue inducement to take risks.

Among representatives from endemic communities, Mr. Asuman Muwumuza, Councillor for Koome sub-county which comprises island communities in Lake Victoria, expressed strong support for the development of the CHI-S in Uganda. He assured the Meeting that local communities would understand the purpose of the study and want to participate, would gladly volunteer and would do whatever would be needed to facilitate these complicated trials. He felt that the need for a vaccine for schistosomiasis was urgent and urged the research community not to delay.

## Ethical and regulatory considerations for CHI-S in Uganda

The fundamental ethical issue of concern in relation to CHI models is the principle of non-maleficence, to do no harm: CHI models represent a new ethical challenge and dilemma – using harm with a view to achieving benefit. Historical atrocities involving deliberate infection of vulnerable populations have an important influence on thinking in this field. Guidelines governing the implementation of CHI models are not available in African countries. While guidelines would be desirable, these take a long time to be developed and approved. At the stakeholders’ meeting, it was recommended that the principles articulated by the World Health Organisation (2016) ^34^ and benchmarks developed at the Malawi meeting on Controlled Human Infection Models in Low Income Countries ^8^ be employed to govern the ethical and regulatory approval process. These are set out in Table 2, which also identifies ways in which the Uganda CHI-S will address them. Among the benchmarks outlined, critical elements discussed included the following. First, ethical and regulatory standards governing CHI studies in Africa must be equivalent to, or above, the minimum human protection standards applied internationally, as well as locally. When necessary, the capacity of ethical and regulatory bodies must be built, as well as the capacity of researchers. Second, risks must be examined and evaluated before considering possible benefits; there must be a favourable benefit: risk ratio. Arguably the risk associated with a controlled human infection may be more justifiable in an endemic population than in an unaffected population. Third, all stakeholders must be fully informed; in particular, as discussed above, volunteers must fully understand the study, its risks, and benefits, and must be shown to do so. Contributions of social science research to identifying ways of achieving this were desirable. 

**Table 2.** Benchmarks identified in the Malawi framework, and approach to addressing them for the Uganda controlled human infection model for *Schistosoma mansoni* (CHI-S). DSMB - data and safety monitoring board.

	Malawi framework benchmarks		Uganda CHI-S
**1**	**Issue of national importance**, within the research agenda	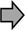	✓ Over half of Uganda’s population estimated to be at risk from schistosomiasis; vaccine development research supported by Vector Control Division (VCD), Ministry of Health
**2**	**Safety** already demonstrated	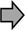	✓ Safety data from Leiden trials ✓ Risk assessments to Uganda to be developed
**3**	Model **quality** established by published data	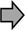	✓ Publication of Leiden trials expected in 2018
**4**	**Strong scientific case**, without alternative approach	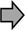	✓ Model has potential to fast-track selection of best vaccine candidates accelerating development of safe, effective vaccines ✓ Available animal models may not determine correlates of protection and vaccine efficacy in humans ✓ Understanding and data needed regarding differences between endemic and non-endemic populations in response to candidate vaccines
**5**	Promotes **capacity development** in country	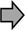	✓ CHI-S preparatory activities already providing opportunities for learning and debate for researchers, ethicists and regulators; continuing interaction between researchers and regulators is planned ✓ Further developments to include relevant infrastructure development and technical training of Ugandan researchers
**6**	**Ethical acceptability** including issues of understanding consent	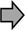	✓ Issues of understanding and voluntariness recognised and to be assured by pilot work in target populations in preparation for CHI-S
**7**	**Governance** structure in place (DSMB, sponsor)	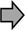	✓ Protocol to be developed with due attention to these requirements

**Table 3.** Establishing a controlled human schistosome infection model in Uganda: key recommendations and next steps. (CHI-S) - Controlled human infection model for *Schistosoma mansoni*, (GCLP) - Good Clinical Laboratory Practice.

Technical steps	
Managing and shedding snails in Uganda	• Establish GCLP level facility for housing and shedding snails in Uganda • Obtain accreditation of facility
Identifying male cercariae and preparing inoculum	• Training Uganda team in technical and quality control and quality assurance procedures
Detection and quantification of schistosome infection in Uganda	• Implementation of the highly sensitive CAA assay in Uganda
Shipping infected snails to Uganda	• Risk assessment regarding environmental contamination • Implementation of risk management measures • Implementation of IATA shipping requirements • Ensuring Material Transfer Agreements are in place prior to shipment • Planning for efficient release by customs officials and handling agents on arrival
Role of endemic CHI-S	• Liaise with vaccine developers to position endemic CHI-S in the vaccine development pipeline
Community and participant recruitment steps	
Community engagement	• Raise awareness for CHI in local communities to ensure understanding and support • Identify engaged communities who would be willing to participate • Include details of planned community engagement (from parliament to local council) in protocol; undertake further preparatory engagement activities
Informed consent	• With social science support, develop tools to ensure and document full understanding by participants • Evaluate the informed consent process and assess the understanding of the study procedures
Management of natural exposure	• Determine feasibility for potential participants of avoiding natural exposure to *S. mansoni* Infection during participation in the challenge model
Ethical and regulatory steps	
Regulatory capacity building	• Provide further information for ethicists and regulators and ethicists through visits to the Leiden facilities • Liaise with African ethicists with previous CHI experience
CHI-S protocol for Uganda	• Draft protocol; pre-submission discussions with regulatory authorities
CHI-S product dossier	• Development of CHI-S product dossier and related documentation for Uganda

In terms of the regulatory landscape, key stakeholders in Uganda include the Uganda National Council for Science and Technology (UNCST), the National Drug Authority (NDA) and National Environment Management Agency (NEMA) as well as Institutional Review Boards. The roles of these authorities were discussed and it was concluded that the UNCST would hold overall authority for approval of importation of snails (infected or otherwise) and for review and approval of a CHI-S protocol. A joint review meeting, with all regulatory authorities represented, was recommended, as well as engagement between the researchers and ethical and regulatory review bodies throughout the process of protocol development and implementation. 

The nature of the human challenge product, the inoculum of infectious cercariae, was noted to present a particular dilemma. Whereas the product for malaria is being investigated under a FDA investigational new drug application ^35^, the CHI-S product must be generated locally for each infection. This requires local laboratory capacity for high-quality production on-site, and local regulatory capacity for approval of the facilities and processes. Under these circumstances, provision of documentation corresponding to the standard requirements of investigator’s brochure, certificate of good manufacturing practice, sample label, certificate of analysis and letter of authorisation from the product “owner” may be difficult, but a product dossier containing equivalent information will be needed. This would be considered alongside full documentation of procedures and results from Leiden by the regulatory bodies. 

## Conclusion and next steps

Researchers, community members and regulators participating in the stakeholders’ meeting expressed substantial support for establishing CHI-S in Uganda; this was considered both feasible and desirable. 

Key next steps ( Table 3) include risk assessments for importation of infected snails, the development of facilities and expertise for production of the challenge product; community engagement and pilot studies to assess information and consent tools and comprehension by target communities, and to define appropriate populations (able to avoid re-infection, and to participate with full understanding and as true volunteers); provision of opportunities for regulators and ethicists to learn more about CHI-S through visits to Leiden and engagement with their Dutch counterparts; and development of a draft CHI-S protocol, product dossier and accompanying documentation for regulatory review.

## Data availability

No data is associated with this article.
