# Supplementary material for: Ethical and scientific considerations on the establishment of a controlled human infection model for schistosomiasis in Uganda: report of a stakeholders’ meeting held in Entebbe, Uganda
Source: AAS Open Res. 2018 Aug 6;1:2. Originally published 2018 Apr 18. [Version 2] doi: 10.12688/aasopenres.12841.2 (PMC6358001; doi:10.12688/aasopenres.12841.2)
Supplement: Peer review history

# Peer review: Reviewer response for version 2

**DOI:** 10.21956/aasopenres.13967.r26567

**Published:** 2018-08-10

**Authors:** Matthew B. Laurens

**Affiliations:** 1 Division of Malaria Research, Institute for Global Health, University of Maryland School of Medicine, Baltimore, MD, USA

The authors have responded adequately and appropriately to all comments and questions.

I confirm that I have read this submission and believe that I have an appropriate level of expertise to confirm that it is of an acceptable scientific standard.

# Peer review: Reviewer response for version 1

**DOI:** 10.21956/aasopenres.13907.r26338

**Published:** 2018-05-15

**Authors:** Matthew B. Laurens

**Affiliations:** 1 Division of Malaria Research, Institute for Global Health, University of Maryland School of Medicine, Baltimore, MD, USA

The open letter by AM Elliott et al. is a well-written, concise meeting summary following a stakeholders' meeting in Entebbe, Uganda, where key individuals discussed relevant issues in establishing a controlled human infection model for *Schistosoma mansoni* in Uganda. The report also reviews the disease burden, biology, and epidemiology of schistosomiasis. A brief update on current vaccines for schistosomiasis is also included. The authors condense outcomes of the meeting into tabular format and relevant next-steps to advance controlled human infection models for schistosomiasis in Uganda.

 Comments:

1. The introduction highlights development of the *S. mansoni *CHI model, but does not completely describe if this model could potentially inform development of a vaccine against *S. mansoni *alone, a model upon which *S. hematobium *and other species could be built, or if a species-transcending vaccine is feasible given the current state of scientific knowledge as proposed by M Merrifield in *Vaccine *2016 article ^1^.
2. The section on epidemiology of **schistosomiasis could be augmented if details on age-specific carriage rates in communities is available, and/or data regarding special populations hardest-hit by schistosomiasis (immunocompromised, children, etc) can be provided. This would orient the reader toward where vaccine efforts are most warranted.
3. The background section on schistosomiasis does not completely discuss *S. hematobium *complications, presumably because the focus of the article is on *S. mansoni*. Consider that a successful model for *S. mansoni *could pave the way for a successful CHI model for *S. hematobium*.
4. The section on vaccines for schistosomiasis notes that an ideal vaccine would be suitable for use among young children. A very brief explanation as to why this population is targeted would be informative for readers.
5. Additional considerations for Table 1 is that schistosomiasis epidemiology is dynamic and may be difficult to predict, such that large field studies testing candidate vaccines may not show effect if significant changes in local epidemiology occur. Also, to study infection prevention as a vaccine trial endpoint in a population, individuals would require pre-treatment before vaccination, and this pre-treatment may decrease community transmission significantly such that vaccine efficacy would be difficult to measure. CHI also allows efficient testing of vaccine efficacy in individuals with a known exposure profile and background immunity to schistosomiasis.
6. The section on endpoints in CHI studies- is parallel testing with Leiden and Uganda planned for the PCR protein detection, or is another backup method to confirm the presence of *S. mansoni *in place? This would be advantageous to address from a quality management perspective.
7. In addition to community engagement plans, careful risk management strategies for CHI in low and middle-income countries are an important consideration as information management and mitigation needs to be planned and ready to activate to mitigate undesired publicity of CHI on social media and other avenues of communication.
8. Are there technical or infrastructure aspects of the research milieu in Uganda that made it suitable as the first African host country for CHI for schistosomiasis? If yes, consider noting these in case other groups are considering pilot CHI studies in low and middle-income countries.

I confirm that I have read this submission and believe that I have an appropriate level of expertise to confirm that it is of an acceptable scientific standard.

## References

[1] : Advancing a vaccine to prevent human schistosomiasis. *Vaccine*.2016;34(26) : 10.1016/j.vaccine.2016.03.079 2988-2991 10.1016/j.vaccine.2016.03.079 27036511

# Response

**Published:** 2018-07-25

**Authors:** Alison Elliott

**Affiliations:** Uganda Virus Research Institute, Uganda

*Comments: *

1. *The introduction highlights development of the S. mansoni CHI model, but does not completely describe if this model could potentially inform development of a vaccine against S. mansoni alone, a model upon which S. hematobium and other species could be built, or if a species-transcending vaccine is feasible given the current state of scientific knowledge as proposed by M Merrifield in Vaccine 2016 article* *^1^* *.*

We have added two sentences on the possibility of developing a S. hematobium CHI based on the S. mansoni blueprint trial. We feel that speculating on the feasibility of strain-transcending vaccines is beyond the scope of the current paper, but have referred to the need to evaluate the requirement for a hematobium model in the clinical development context.

1. *The section on epidemiology of schistosomiasis could be augmented if details on age-specific carriage rates in communities is available, and/or data regarding special populations hardest-hit by schistosomiasis (immunocompromised, children, etc) can be provided. This would orient the reader toward where vaccine efforts are most warranted.*

We have added some further information in this regard. 

1. *The background section on schistosomiasis does not completely discuss S. hematobium complications, presumably because the focus of the article is on S. mansoni. Consider that a successful model for S. mansoni could pave the way for a successful CHI model for S. hematobium.*

Please refer to the adaptations made in response to comment 1: we have added the possibility of an S. hematobium model to the manuscript.

1. *The section on vaccines for schistosomiasis notes that an ideal vaccine would be suitable for use among young children. A very brief explanation as to why this population is targeted would be informative for readers.*

We have added a note that children would be targeted given their high burden of infection  

1. *Additional considerations for Table 1 is that schistosomiasis epidemiology is dynamic and may be difficult to predict, such that large field studies testing candidate vaccines may not show effect if significant changes in local epidemiology occur. Also, to study infection prevention as a vaccine trial endpoint in a population, individuals would require pre-treatment before vaccination, and this pre-treatment may decrease community transmission significantly such that vaccine efficacy would be difficult to measure. CHI also allows efficient testing of vaccine efficacy in individuals with a known exposure profile and background immunity to schistosomiasis.*

We have added these considerations to table 1.

1. *The section on endpoints in CHI studies- is parallel testing with Leiden and Uganda planned for the PCR protein detection, or is another backup method to confirm the presence of S. mansoni in place? This would be advantageous to address from a quality management perspective.*

Validation of such critical assays (as well as the CAA assay) would of course be essential to ensure the same quality in Uganda and Leiden. Thorough validation as well as quality control measures will be put in place to ensure this. Parallel testing could be part of this programme, but rather an exchange of control samples with known outcomes would be more likely. We have added a remark on this important point.

 

1. *In addition to community engagement plans, careful risk management strategies for CHI in low and middle-income countries are an important consideration as information management and mitigation needs to be planned and ready to activate to mitigate undesired publicity of CHI on social media and other avenues of communication.*

We have added this point to the section on community engagement.

1. *Are there technical or infrastructure aspects of the research milieu in Uganda that made it suitable as the first African host country for CHI for schistosomiasis? If yes, consider noting these in case other groups are considering pilot CHI studies in low and middle-income countries.*

We thank the reviewer for this suggestion.  We have added a short paragraph on this under the new heading “Considerations on implementation of the novel CHI-S model in the endemic setting in Uganda”

# Peer review: Reviewer response for version 1

**DOI:** 10.21956/aasopenres.13907.r26337

**Published:** 2018-05-02

**Authors:** Sean C. Murphy

**Affiliations:** 1 Department of Laboratory Medicine, University of Washington, Seattle, WA, USA

The report by Elliott *et al.* provides a well-written summary of schistosomiasis and the controlled human infection (CHI) model for Schistosoma (CHI-S) developed by the Leiden team followed by an integrated report about a stakeholders meeting conducted in Uganda ahead of possible implementation of the CHI-S model in the endemic Uganda-based site.  

**Major issues:**

- While the paper provides an excellent overview of schistosomiasis and the CHI-S model, the transition from background on schistosomiasis to the report on the meeting could benefit from a brief foreshadowing of the major findings from the stakeholders meeting.  While the technical hurdles to implementation of the CHI-S model in Uganda were more easily identified and labeled (and are present in Table 3), it seems more difficult to identify discrete core ethical and community based hurdles.  The authors could consider adding more bullet points to Table 3 to expand on some of these ethical and community based hurdles.  The authors could also add a few overview-type sentences when the paper transitions from Background/Summary to Stakeholder meeting report.

**Minor issues:**

- The section titled “The controlled human schistosome infection model” could be re-titled “The controlled human schistosome infection model and considerations for adaptation to Ugandan site”.
- Is there any possibility that adoption of new strains could carry unexpected bacterial flora that could cause unexpected AEs?
- The scientific rationale for importation of the Leiden CHI-S snails/parasites as compared to use of locally-acquired snails/parasites could be expanded upon more fully.
- The following sentence is *much *too long and should be split into 2-3 sentences to improve readability: “Populations of interest for CHI-S will include Ugandans not previously exposed to schistosomiasis (perhaps from an urban setting) as well as those from schistosomiasis-endemic communities (prior exposure for inclusion or exclusion can be determined by measuring IgG antibody to schistosome egg antigen): experience in Kenya with the controlled human malaria infection (CHMI) model showed that participants coming from areas with no active transmission (Nairobi residents) had low baseline responses to malaria, and a challenge response similar to Europeans3, whereas those resident where active malaria transmission occurs had higher baseline responses - indicative of either recent or prior malaria exposure - and a distinct profile of response to challenge (Kapulu, Bejon personal communication)."
- Note that Sanaria's PfSPZ product is NOT yet "FDA approved".  This misunderstanding should be corrected toward the end of the section titled “Ethical and regulatory considerations for CHI-S in Uganda”.  The Sanaria product is being investigated under an FDA Investigational New Drug application.
- The section “Contribution from representatives of endemic communities” lists contributions from a single individual who is said to speak for some of these communities.  However, the section title sounds more generalized than the text indicates.  I would suggest folding this section into another section as the current representation seems to potentially overly generalize the responses from one (albeit generous and collaborative) individual.

I confirm that I have read this submission and believe that I have an appropriate level of expertise to confirm that it is of an acceptable scientific standard.

# Response

**Published:** 2018-07-25

**Authors:** Alison Elliott

**Affiliations:** Uganda Virus Research Institute, Uganda

***Major issues:***

- *While the paper provides an excellent overview of schistosomiasis and the CHI-S model, the transition from background on schistosomiasis to the report on the meeting could benefit from a brief foreshadowing of the major findings from the stakeholders meeting.  While the technical hurdles to implementation of the CHI-S model in Uganda were more easily identified and labeled (and are present in Table 3), it seems more difficult to identify discrete core ethical and community based hurdles.  The authors could consider adding more bullet points to Table 3 to expand on some of these ethical and community based hurdles.  The authors could also add a few overview-type sentences when the paper transitions from Background/Summary to Stakeholder meeting report.*

We have added the requested bullet points to Table 3 to expand on the ethical and community based hurdles. In addition, we have added an introductory paragraph where the paper transitions from describing the background to the findings in the stakeholders meeting, to ensure that the switch is clear to the readers.

***Minor issues:***

- *The section titled “The controlled human schistosome infection model” could be re-titled “The controlled human schistosome infection model and considerations for adaptation to Ugandan site”.*

We agree with the reviewer suggestion to make clear that we will specifically address the Ugandan site. However, taking into account also the major issue raised by this reviewer that the transition of the manuscript from introduction to the findings of the meeting was unclear, we have decided to do this by adding a section title “ **Considerations on implementation of the novel CHI-S model in the endemic setting in Uganda**” to mark the transition of the paper and simultaneously indicate the specific relevance to Uganda.

- *Is there any possibility that adoption of new strains could carry unexpected bacterial flora that could cause unexpected AEs?*

Infected snails are tested for their bioburden before cercaria are used for CHI-S purpose. Potentially new strains could carry a different bioburden which would preclude their use in CHI-S, but this needs to be tested. To highlight the fact that bioburden testing would need to take place for new strains we have added this in our section on technical considerations.

- *The scientific rationale for importation of the Leiden CHI-S snails/parasites as compared to use of locally-acquired snails/parasites could be expanded upon more fully.*

We have expanded the requested paragraph.

- *The following sentence is much too long and should be split into 2-3 sentences to improve readability: “Populations of interest for CHI-S will include Ugandans not previously exposed to schistosomiasis (perhaps from an urban setting) as well as those from schistosomiasis-endemic communities (prior exposure for inclusion or exclusion can be determined by measuring IgG antibody to schistosome egg antigen): experience in Kenya with the controlled human malaria infection (CHMI) model showed that participants coming from areas with no active transmission (Nairobi residents) had low baseline responses to malaria, and a challenge response similar to Europeans3, whereas those resident where active malaria transmission occurs had higher baseline responses - indicative of either recent or prior malaria exposure - and a distinct profile of response to challenge (Kapulu, Bejon personal communication)."*

We have split the sentence.

- *Note that Sanaria's PfSPZ product is NOT yet "FDA approved".  This misunderstanding should be corrected toward the end of the section titled “Ethical and regulatory considerations for CHI-S in Uganda”.  The Sanaria product is being investigated under an FDA Investigational New Drug application.*

This mistake has been corrected.

- *The section “Contribution from representatives of endemic communities” lists contributions from a single individual who is said to speak for some of these communities.  However, the section title sounds more generalized than the text indicates.  I would suggest folding this section into another section as the current representation seems to potentially overly generalize the responses from one (albeit generous and collaborative) individual.*

We have displaced this paragraph so that it falls under the section on “recruitment considerations”
